# Differences in trajectory of disease activity according to biologic and targeted synthetic disease-modifying anti-rheumatic drug treatment in patients with rheumatoid arthritis

**DOI:** 10.1186/s13075-022-02918-3

**Published:** 2022-10-14

**Authors:** Bon San Koo, Seongho Eun, Kichul Shin, Seokchan Hong, Yong-Gil Kim, Chang-Keun Lee, Bin Yoo, Ji Seon Oh

**Affiliations:** 1 Department of Internal Medicine, Inje University Seoul Paik Hospital, Inje University College of Medicine, Seoul, South Korea; 2 Department of Management Engineering, College of Business, KAIST, Seoul, South Korea; 3 Division of Rheumatology, Seoul Metropolitan Government-Seoul National University Hospital Boramae Medical Center, Seoul, South Korea; 4 Division of Rheumatology, Department of Internal Medicine, Asan Medical Center, University of Ulsan College of Medicine, Seoul, South Korea; 5 Department of Information Medicine, Big Data Research Center, Asan Medical Center, Seoul, South Korea

**Keywords:** Rheumatoid arthritis, Biologics, Trajectory clustering/trajectory modeling, Treatment response

## Abstract

**Background:**

The purpose of this study was to stratify patients with rheumatoid arthritis (RA) according to the trend of disease activity by trajectory-based clustering and to identify contributing factors for treatment response to biologic and targeted synthetic disease-modifying anti-rheumatic drugs (DMARDs) according to trajectory groups.

**Methods:**

We analyzed the data from a nationwide RA cohort from the Korean College of Rheumatology Biologics and Targeted Therapy registry. Patients treated with second-line biologic and targeted synthetic DMARDs were included. Trajectory modeling for clustering was used to group the disease activity trend. The contributing factors using the machine learning model of SHAP (SHapley Additive exPlanations) values for each trajectory were investigated.

**Results:**

The trends in the disease activity of 688 RA patients were clustered into 4 groups: rapid decrease and stable disease activity (group 1, *n* = 319), rapid decrease followed by an increase (group 2, *n* = 36), slow and continued decrease (group 3, *n* = 290), and no decrease in disease activity (group 4, *n* = 43). SHAP plots indicated that the most important features of group 2 compared to group 1 were the baseline erythrocyte sedimentation rate (ESR), prednisolone dose, and disease activity score with 28-joint assessment (DAS28) (SHAP value 0.308, 0.157, and 0.103, respectively). The most important features of group 3 compared to group 1 were the baseline ESR, DAS28, and estimated glomerular filtration rate (eGFR) (SHAP value 0.175, 0.164, 0.042, respectively). The most important features of group 4 compared to group 1 were the baseline DAS28, ESR, and blood urea nitrogen (BUN) (SHAP value 0.387, 0.153, 0.144, respectively).

**Conclusions:**

The trajectory-based approach was useful for clustering the treatment response of biologic and targeted synthetic DMARDs in patients with RA. In addition, baseline DAS28, ESR, prednisolone dose, eGFR, and BUN were important contributing factors for 4-year trajectories.

## Background

Patients with rheumatoid arthritis (RA) suffer from chronic inflammatory arthritis and a variety of extra-articular symptoms. Because RA patients develop long-term disabilities and decreased quality of life, they need intensive treatment early on, including treat-to-target strategies [1, 2]. Second-line disease-modifying anti-rheumatic drugs (DMARDs), such as biologic DMARDs (bDMARDs) or targeted synthetic DMARDs (tsDMARDs), are used in the treatment of RA patients who do not respond to treatment with conventional synthetic DMARDs (csDMARDs) [3–5]. However, because individual patients respond differently to bDMARDs or tsDMARDs, selecting the drug that will induce the best response in each patient remains challenging in clinical practice. Indeed, finding predictors of optimal treatment response to second-line DMARDs is necessary not only to improve the prognoses, but also to reduce patient suffering and medical costs [6, 7].

Although various predictors for treatment response have been proposed thus far [8–11], most studies have evaluated the treatment response at a specific time point (e.g., 3 or 6 months) without considering the changes in disease activity over time in the predictive models. Among patients treated with bDMARDs or tsDMARDs, some show improvements in disease activity early during treatment, while others respond slowly to treatment and their prescription is either stopped or switched to another drug. In addition, even if the initial response to treatment is good, some patients show aggravation of disease activity over time, and some patients do not improve disease activity even if the biological agent is maintained or switched. Given that changes can vary over a relatively long period of time, it is important to evaluate long-term treatment responses and to consider disease activity at multiple time points.

Trajectory-based clustering is a group-based approach that has been recently used in several studies on patients with RA [12–20]. This method can determine the clusters of individuals with a similar course of disease activity and progression over time and identify different patterns of response to treatment. Using predictive markers that are associated with each distinct disease activity pattern can increase the efficiency of the treatment response and maintain treatment effects for a long time.

The purpose of the present study was twofold: (1) to investigate trajectories by classifying long-term changes in treatment response to bDMARDs or tsDMARDs in patients with RA and (2) to find contributing factors in each trajectory using machine learning models.

## Methods

### Study population and data collection

This study used data from the KOBIO registry, a nationwide multicenter cohort in Korea that was established to evaluate the effectiveness and side effects of bDMARDs (abatacept, adalimumab, etanercept, golimumab, infliximab, rituximab, and tocilizumab) and tsDMARDs (tofacitinib and baricitinib) in patients with RA [21]. Patients in the registry were recruited from 38 hospitals since 2012, and their demographics, medications, comorbidities, extra-articular manifestations, disease activities, radiographic findings, and laboratory findings have been recorded by investigators from each participating hospital. A total of 2,122 patients who were treated with second-line DMARDs including bDMARDs and tsDMARDs between December 2012 and June 2019, were registered. Patients who had been treated with bDMARDs or tsDMARDs before registration in the KOBIO registry were excluded from the study. The number of patients and number of follow-ups that each patient completed, were counted to find the most appropriate follow-up period for clustering. Patients who completed four follow-ups were selected. Patients who did not complete four follow-ups due to various reasons, including refusal of further registration, loss of follow-up, or less than four years of follow-up, were excluded from the study. Ethical approval of the KOBIO registry was obtained from the institutional review boards of all 38 participating institutions, including the Institutional Review Board of Inje University Seoul Paik Hospital (PAIK 2018–11-005).

### Trajectory-based clustering model

Group-based trajectory modeling (GBTM) is a model for clustering sequential data and has often been used in clinical studies [22]. The purpose of GBTM is to find groups with different characteristics by clustering response patterns over time and to compare the clustered groups. The model assumes that the dependent variable follows a certain probability distribution such as normal distribution or Poisson distribution and that the mean of the probability distribution takes the form of polynomials over time. Under the above assumptions, parameters and functional forms are fitted by the data using maximum likelihood estimation. In our study, the sequential treatment records using biologics were clustered by response pathway based on the model, and the probability distribution and parameters were determined by the medical rationale and data explanatory power. The probability of distribution of the dependent variable was chosen as the censored normal distribution. The number of clusters and the order of polynomials were determined to maximize the value of the model selection criterion, Bayesian Information Criterion, by performing several combinations. After clustering, we showed the individual response patterns to confirm the empirical results. Group-based trajectory modeling was performed using Stata software.

### Machine learning model for finding contributing factors of trajectories

A machine learning model was built to find clustered groups and contributing factors. Clustered groups were used as outputs and potential important factors as inputs, and the contribution of each variable was evaluated. First, an XGBoost (eXtreme Gradient Boosting) model was employed to fit a prediction function whose output represented a long-term clinical path [23]. We used an XGBoost as this is a tree-based ensemble model known for its high performance in many machine learning prediction tasks. To determine the degree of the added variable contribution to the prediction in the model, we used a SHAP (SHapley Additive exPlanations) value, an additive feature attribution method used for interpretability when building complex models for high prediction power such as ensemble models and deep learning [24]. By using both XGBoost and the SHAP value, we were able to identify clinical parameters that contributed to the differentiation of poor and good response pathways and thus, had potential clinical implications. XGBoost and SHAP values were estimated using R software version 3.6.1 (R Foundation for Statistical Computing, Vienna, Austria).

### Statistical analysis

For comparison of baseline characteristics among groups, the chi-squared and Kruskal–Wallis tests were used for categorical and continuous variables, respectively. Statistical analyses were performed using R software. All data are shown as mean (standard deviation [SD]) or percentage values.

## Results

### Patient selection and grouping according to disease trajectory

Among the patients treated with tsDMARDs, only those treated with tofacitinib were included in the study. Of the 2122 patients, we excluded 353 patients treated with bDMARDs or tsDMARDs prior to registration in the KOBIO registry, and 66 patients with missing data. Among 1703, the second, third, fourth, fifth, sixth, and seventh follow-ups were 1307, 959, 688, 454, 217, and 61, respectively. Of the 1703 patients, 688 completed four follow-ups and therefore, were selected for clustering.

**Fig. 1:**
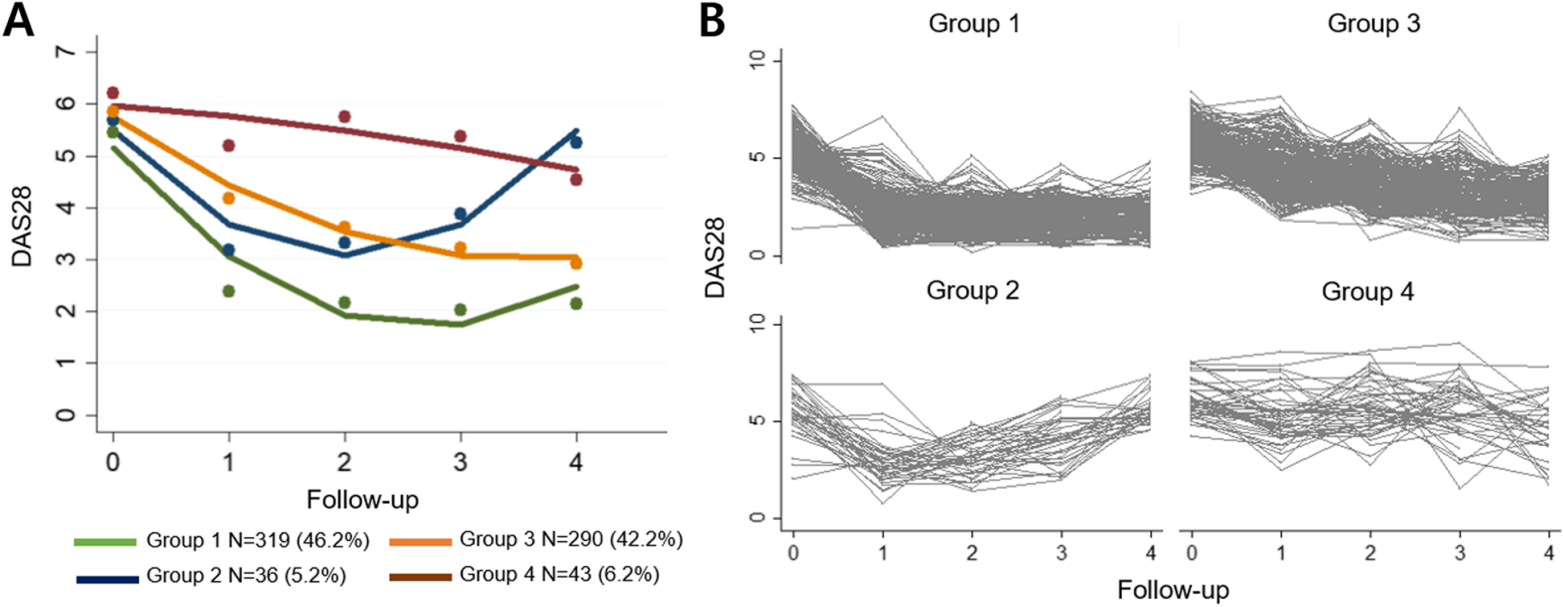
Trajectory-based clustering. Trajectory clustered into 4 groups (**A**) and changes in the disease activities of each individual over 4 years (**B**). Abbreviation: DAS28, disease activity scores with 28-joints assessment using the erythrocyte sedimentation rate

The 688 study patients were allocated to 4 distinct groups according to the trajectory of DAS28 (Fig. 1). In group 1 (*n* = 319), the disease activity rapidly decreased and remained below moderate; in group 2 (*n* = 36), the disease activity rapidly decreased but then increased; in group 3 (*n* = 290), the disease activity showed a continuous but slow decrease; and in group 4 (*n* = 43), the disease activity remained high. The trajectories of each individual patient are shown in Fig. 1B. The baseline characteristics, including the number of current smokers, BUN, cholesterol, and initial DAS28, differed significantly among the groups (Table 1).

**Table 1:** Baseline characteristics of patients according to trajectory-based clustering

	**Total** **(*N* = 688)**	**Group 1** **(*N* = 319)**	**Group 2** **(*N* = 36)**	**Group 3** **(*N* = 290)**	**Group 4** **(*N* = 43)**	***p***
Age, year	52.9 (12.2)	52.0 (12.3)	53.6 (13.2)	53.6 (12.1)	54.4 (11.1)	0.324
Male sex, *n* (%)	97 (14.1)	46 (14.4)	7 (19.4)	35 (12.1)	9 (20.9)	0.318
Body mass index, kg/m^2^	22.4 (3.4)	22.2 (2.9)	22.5 (2.7)	22.7 (3.8)	22.4 (4.2)	0.723
Symptom duration, year	7.2 (7.1)	7.1 (7.2)	8.3 (8.4)	7.2 (6.9)	7.6 (7.5)	0.925
Smoking, *n* (%)						
Current smoker	38 (5.5)	11 (3.4)	1 (2.8)	20 (6.9)	6 (14.0)	0.018
Ex-smoker	55 (8.0)	28 (8.8)	3 (8.3)	19 (6.6)	5 (11.6)	0.601
Never smoker	595 (86.5)	280 (87.8)	32 (88.9)	251 (86.6)	32 (74.4)	0.112
Prednisolone dose*, mg	5.1 (3.7)	4.9 (3.7)	5.9 (6.4)	5.1 (3.2)	5.2 (3.6)	0.570
Methotrexate dose, mg	10.5 (5.6)	10.6 (5.3)	11.3 (4.9)	10.3 (5.8)	9.3 (6.7)	0.885
Hemoglobin, g/dL	12.0 (1.4)	12.2 (1.3)	12.0 (1.1)	11.9 (1.4)	11.6 (1.5)	0.014
ALT, IU/L	19.2 (12.5)	19.2 (11.6)	20.1 (12.4)	19.0 (12.3)	20.0 (18.5)	0.393
AST, IU/L	21.2 (9.8)	20.9 (7.1)	21.6 (6.9)	21.3 (9.0)	23.5 (24.1)	0.463
BUN, mg/dL	14.2 (4.5)	14.5 (4.5)	14.0 (4.3)	13.7 (4.2)	15.6 (5.9)	0.118
eGFR, mL/min/1.732m^2^	101.2 (26.7)	101.2 (26.9)	104.1 (26.4)	101.2 (26.9)	97.9 (25.1)	0.702
Cholesterol, mg/dL	174.3 (27.7)	171.4 (27.0)	171.1 (22.0)	178.6 (28.7)	169.0 (26.5)	0.054
ESR, mm/h	49.2 (25.4)	43.0 (22.9)	54.6 (31.7)	54.5 (25.2)	55.6 (28.8)	< 0.001
CRP, mg/dL	2.2 (2.8)	2.1 (2.4)	2.7 (2.8)	2.4 (3.2)	2.0 (2.1)	0.376
ANA positivity, *n* (%)	247 (35.9)	116 (36.4)	13 (36.1)	104 (35.9)	14 (32.6)	0.971
RF positivity, *n* (%)	595 (86.5)	275 (86.2)	32 (88.9)	255 (87.9)	33 (76.7)	0.240
ACPA positivity, *n* (%)	515 (74.9)	238 (74.6)	27 (75.0)	220 (75.9)	30 (69.8)	0.859
Initial DAS28	5.68 (0.95)	5.4 (0.9)	5.5 (1.2)	5.9 (0.9)	6.1 (1.0)	< 0.001

Data are mean (standard deviation) unless noted otherwise

*ACPA*, anti-cyclic citrullinated peptide antibody; *ALT*, alanine aminotransferase; *ANA*, anti-nuclear antibody; *AST*, aspartate aminotransferase; *BUN*, blood urea nitrogen; *CRP*, C-reactive protein; *DAS28*, disease activity score with 28-joint assessment; *eGFR*, estimated glomerular filtration rate; *ESR*, erythrocyte sedimentation rate; *RF*, rheumatoid factor

^*^Glucocorticoid dose (e.g., prednisolone, methylprednisolone, deflazacort, and dexamethasone) was converted to prednisolone doses

### Prescription patterns of bDMARDs and tsDMARDs according to trajectory groups

Table 2 shows the prescription patterns of bDMARDs and tsDMARDs according to trajectory groups. The proportion of patients who maintained their first prescribed bDMARDs or tsDMARDs during the follow-up period (never switching) in group 1 was 86.5%, which was numerically higher than those in groups 2, 3, and 4 (52.8%, 50.3%, and 25.6%, respectively). The mean number of bDMARDs or tsDMARDs prescribed was numerically higher in group 4 (2.3) than in groups 1 (1.1), 2 (1.7), and 3 (1.6). The proportion of patients who were prescribed non-tumor necrosis factor (TNF) inhibitor as initial treatment was numerically higher in group 1 (44.2%) than in groups 2, 3, and 4 (30.5%, 23.4%, and 18.6%, respectively).

**Table 2:** Switching pattern of bDMARDs or tsDMARDs in each trajectory group

	**Group 1** **(*N* = 319)**	**Group 2** **(*N* = 36)**	**Group 3** **(*N* = 290)**	**Group 4** **(*N* = 43)**
Number of switches for bDMARDs or tsDMARDs, *n* (%)				
Never	276 (86.5)	19 (52.8)	146 (50.3)	11 (25.6)
Once	40 (12.5)	11 (30.6)	115 (39.7)	14 (32.6)
Twice	3 (0.9)	5 (13.9)	26 (9.0)	11 (25.6)
Three times	0 (0.0)	1 (2.8)	2 (0.7)	6 (14.0)
Four times	0 (0.0)	0 (0.0)	1 ( 0.3)	1 ( 2.3)
Number of bDMARDs or tsDMARDs, mean (SD)	1.1 (0.4)	1.7 (0.8)	1.6 (0.7)	2.3 (1.1)
Number of TNF inhibitors, mean (SD)	0.6 (0.5)	1.0 (0.8)	0.9 (0.6)	1.2 (0.7)
Number of non-TNF inhibitors, mean (SD)	0.6 (0.6)	0.7 (0.6)	0.7 (0.7)	1.2 (1.0)
Initial bDMARDs or tsDMARDs, *n* (%)				
Adalimumab	56 (17.6)	11 (30.6)	76 (26.2)	8 (18.6)
Etanercept	60 (18.8)	7 (19.4)	57 (19.7)	14 (32.6)
Golimumab	21 (6.6)	1 (2.8)	28 (9.7)	3 (7.0)
Infliximab	41 (12.9)	6 (16.7)	61 (21.0)	10 (23.3)
Abatacept	47 (14.7)	3 (8.3)	39 (13.4)	1 (2.3)
Rituximab	0 (0.0)	0 (0.0)	1 (0.3)	0 (0.0)
Tocilizumab	93 (29.2)	8 (22.2)	28 (9.7)	6 (14.0)
Tofacitinib	1 (0.3)	0 (0.0)	0 (0.0)	1 (2.3)
Initial TNF inhibitor or non-TNF inhibitor, *n* (%)				
TNF inhibitor	178 (55.8)	25 (69.4)	222 (76.6)	35 (81.4)
Non-TNF inhibitor	141 (44.2)	11 (30.5)	68 (23.4)	8 (18.6)

*bDMARDs*, biologic disease-modifying anti-rheumatic drugs; *TNF*, tumor necrosis factor; *SD*, standard deviation; *tsDMARDs*, targeted synthetic disease-modifying anti-rheumatic drugs

### Contributing factors for trajectory-based clustered groups

**Fig. 2:**
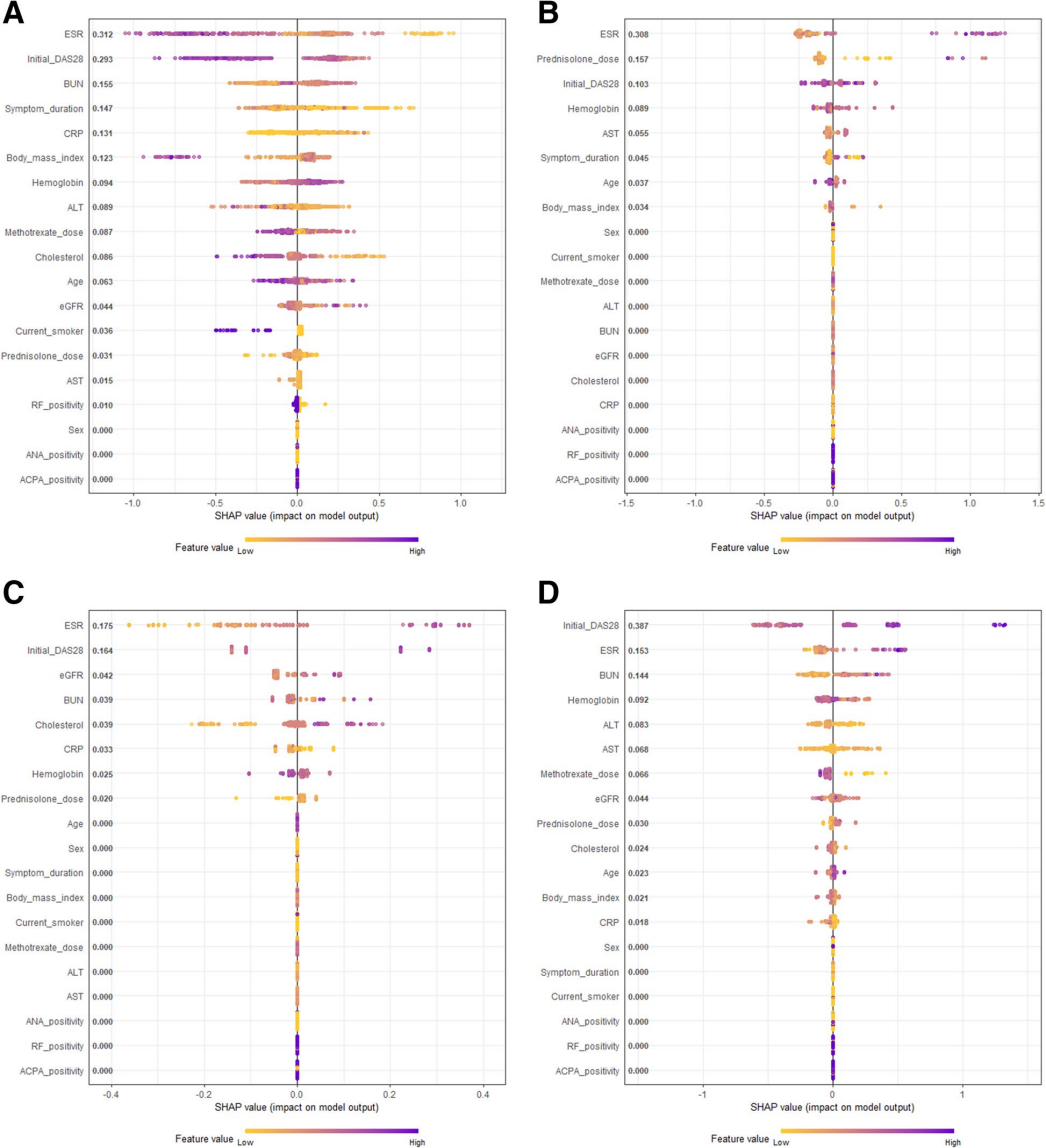
SHAP plots for baseline important features of trajectories. Important features of group 1 compared to groups 2, 3, and 4 (**A**), group 2 compared to group 1 (**B**), group 3 compared to group 1 (**C**), and group 4 compared to group 1 (**D**). Abbreviations: ACPA, anti-cyclic citrullinated peptide antibody; ALT, alanine aminotransferase; ANA, anti-nuclear antibody; AST, aspartate aminotransferase; BUN, blood urea nitrogen; CRP, C-reactive protein; DAS28, disease activity score with 28-joint assessment; eGFR, estimated glomerular filtration rate; ESR, erythrocyte sedimentation rate; RF, rheumatoid factor

Figure 2 shows SHAP plots illustrating the contribution of each of the clinical features to the trajectory-based clustered groups. High SHAP values represent variables that have a high contribution to the trajectory group, either with a positive impact (right side of the zero point on the *x*-axis), or a negative impact (negative values on the *x*-axis); the color (yellow to purple) indicates the feature value, which varies from “low” (yellow) to “high” (purple). Figure 2A indicates the contributing features to the trajectory-based cluster group 1 compared to groups 2, 3, and 4. The baseline ESR was the most important feature (SHAP value 0.312) to the group 1 trajectory, indicating that the lower its feature value (yellow dots, right side), the more ESR related to group 1, and the higher its feature value (purple dots, left side), the less ESR related to group 1. The second important feature was the baseline DAS28 (SHAP value 0.293), which followed a similar pattern to baseline ESR: the higher the DAS28 feature value (purple dots), the less this feature related to group 1, and the lower the feature value (light purple and yellow dots), the more DAS28 was related to group 1. The third important feature was BUN (SHAP value 0.155): the higher it was (light purple dots), the more it was related to groups 2, 3, and 4 (right side).

Figure 2B shows the contributing features of group 2 compared to group 1. The first important feature was the baseline ESR (SHAP value 0.308). The SHAP plot indicates that the higher the ESR feature value, the more ESR was related to group 2. The second important feature was the prednisolone dose (SHAP value 0.157): the higher it was, the more it was related to group 2, although the tendency was not high. The third important feature was baseline DAS28 (SHAP value 0.103), and the median value was related to group 1. Figure 2C shows the important features of group 3 compared to group 1. The baseline ESR was the first important feature (SHAP value 0.175). As indicated in the SHAP plot, the higher the ESR feature value, the more it was related to group 3. The second important feature was the baseline DAS28 (SHAP value 0.164), with a similar pattern as baseline ESR’s contribution to group 3. A third important feature was eGFR (SHAP value 0.042): the higher it was, the more it was related to group 3. Figure 2D shows the important features of group 4 compared to group 1. The first important feature was the baseline DAS28 (SHAP value 0.387): the higher its feature value, the more it was related to group 4. ESR (SHAP value 0.167) and BUN (SHAP value 0.144) followed a similar contribution pattern to baseline DAS28: the higher their feature values, the more they related to group 4. Other variables, such as symptom duration and CRP (Fig. 2A), hemoglobin and AST (Fig. 2B), BUN and cholesterol (Fig. 2C), and hemoglobin and ALT (Fig. 2D) were the fourth and fifth important features.

## Discussion

In this study, we clustered the trajectories of the disease activity of RA patients during 4 years of treatment with bDMARDs or tsDMARDs into 4 patterns. From those trajectories, we found several distinguishing baseline features of 3 trajectories compared with the trajectory with good treatment response (i.e., group 1 with a rapid decrease in disease activity without subsequent increases). Among the baseline features, DAS28 and ESR were common important contributing factors associated with treatment response trajectory in patients with RA treated with bDMARDs or tsDMARDs. In addition, laboratory findings, such as BUN, CRP, hemoglobin, AST, eGFR, cholesterol, and ALT and clinical findings such as prednisolone dose and symptom duration were also high-ranked important features for trajectories.

Various trajectory studies have been conducted on RA patients. A study using the Swedish BARFOT cohort identified three trajectories: best (39.6%), moderate (41.5%), and worst (18.9%) outcomes [13]. Trajectories using Australia’s cohort were similarly divided into good (43.8%), moderate (39.7%), and poor (16.5%) outcome groups and there was a significant difference in BMI and the proportion of ever smokers between the poor outcomes group and the good outcomes group. [16]. A study using the CATCH cohort in Canada divided the cohort into 5 trajectories, which were similar to those of the BARFOT cohort [12]. The strength of our study is that the treatment responses for bDMARDs or tsDMARDs were clustered into 4 trajectories from a 4-year longitudinal cohort data, which is a relatively long period compared with those of other studies.

The latent class mixed model by RA-MAP consortium from the TACERA longitudinal cohort study takes categorical latent variables and assumes each latent class is a function of covariates [18]. On the other hand, group-based trajectory modeling in our study is suitable for classifying trajectories of the dependent variable by assuming that the dependent variable has a polynomial trajectory. Under the assumption that treatment response patterns can be expressed as changes in DAS28, we applied group-based trajectory modeling. In addition to estimating latent classes, important contributing factors of latent classes based on machine learning were provided for clinical implications.

In terms of the switching treatments (bDMARDs or tsDMARDs), as much as 86.5% of patients in group 1 maintained the initially prescribed drug without switching. Moreover, they responded more quickly to treatment with bDMARDs or tsDMARDs than those in other groups, and their disease activity remained low for nearly 4 years. Interestingly, patients in group 1 were more commonly prescribed non-TNF inhibitors as an initial treatment than in those in other groups. In groups 3 and 4, 50.3% and 25.6% of patients maintained one bDMARDs or tsDMARDs, respectively, and 10.0% and 41.9% of patients switched among the DMARDs twice or more, respectively. Differences in trajectories, such as delayed or poor treatment response despite frequent drug replacement, suggest the need for personalized drug selection for each RA patient [25–27]. Moreover, considering the multifactorial characteristics of RA, new drugs countering the various mechanisms of RA must be developed [28, 29].

SHAP value can be expressed as a plot to recognize the interaction of complex predictors [27]. A method of finding predictors of the treatment response of bDMARDs or tsDMARDs that is affected by many variables can help us understand the trajectory of the therapeutic response. In this study, baseline ESR and DAS28 were the most important features in common for predicting trajectory. Interestingly, higher eGFR was associated with group 3 compared to group 1. That is, the higher the renal function, the slower the treatment response may be. In addition, median DAS28 value related to group 2 cannot be captured in a typical regression analysis.

Previous studies have suggested that hemoglobin is associated with disease activity [30, 31]. Our results indicate that hemoglobin may be a contributing factor related to long-term treatment response in some RA patients. Also, cholesterol was the fifth important feature among variables contributing to group 3 compared to group 1. Patients with high baseline cholesterol are unlikely to show a good treatment response [32].

Our study had some limitations. First, we did not consider changes in disease activity related to the type and dosage of DMARDs and individual adherence. Therefore, it may be difficult to generalize the results of this study to individual patients. Second, a small number of tsDMARD users were included in this study, which limits the generalizability of our results. Third, the number of patients and/or duration of the follow-up may have limited the number of trajectories. Indeed, we might discover more than four trajectories if increasing the number of patients and duration of follow-up. Fourth, the inclusion of only patients who completed the fourth follow-up in this study may have a selection bias in which patients who responded well to treatment were primarily selected. In this study, patients who due to a variety of reasons did not complete 4 follow-ups, were excluded. Fifth, important features of the baseline for the trajectory were identified. These important features are potential predictive factors, but statistical verification is required in various studies to confirm they are indeed risk factors.

In conclusion, we were able to cluster the trend of disease activity over 4 years in patients treated with bDMARDs or tsDMARDs into 4 distinct trajectories and found that DAS28, ESR, BUN, CRP, hemoglobin, AST, eGFR, cholesterol, ALT, prednisolone dose, and symptom duration may be important contributing factors for trajectories. Our study suggests that the trajectory-based clustering approach for disease activity may be useful in predicting treatment responses from longitudinal data in real-world practice and making decisions about treatment plans in patients with RA.

## Data Availability

Data are available from the Clinical Research Committee of KOBIO under the Korean College of Rheumatology for researchers who meet the criteria for access to confidential data. To request data, please contact Kichul Shin, MD, PhD, Director of the Korean College of Rheumatology Biologics Registry, Associate Professor of the Division of Rheumatology, Director of Logistics Planning at SMG-SNU, Boramae Medical Center, 20 Boramae-ro-5-gil, Dongjak-gu, Seoul, 07,061, Korea; Tel: + 82–2-870–3204; Fax: + 82–2-870–3866; Email: rk.ca.uns@1bedik.

## Abbreviations

ACPA: Anti-cyclic citrullinated peptide antibody
ALT: Alanine aminotransferase
ANA: Anti-nuclear antibody
AST: Aspartate aminotransferase
bDMARDs: Biologic disease-modifying anti-rheumatic drugs
BUN: Blood urea nitrogen
CI: Confidence interval
CRP: C-reactive protein
DAS28: Disease activity score with 28-joint assessment
DAS28-ESR: Disease activity scores in 28 joints using the erythrocyte sedimentation rate
DMARDs: Disease-modifying anti-rheumatic drugs
eGFR: Estimated glomerular filtration rate
ESR: Erythrocyte sedimentation rate
GBTM: Group-based trajectory modeling
KOBIO: Korean College of Rheumatology Biologics & Targeted therapy Registry
LB: Lower bound
OR: Odds ratio
RA: Rheumatoid arthritis
RF: Rheumatoid factor
SD: Standard deviation
SHAP: SHapley Additive exPlanations
TNF: Tumor necrosis factor
tsDMARDs: Targeted synthetic disease-modifying anti-rheumatic drugs
UB: Upper bound
XGBoost: EXtreme Gradient Boosting
